# Evaluating Outcomes of a Social Media–Based Peer and Clinician-Supported Smoking Cessation Program in Preventing Smoking Relapse: Mixed Methods Case Study

**DOI:** 10.2196/25883

**Published:** 2021-09-20

**Authors:** Naohi Isse, Yuki Tachibana, Makiko Kinoshita, Michael D Fetters

**Affiliations:** 1 Department of General Medicine Ako Municipal Hospital Ako Japan; 2 Department of Internal Medicine Okinawa Prefectural Yaeyama Hospital Ishigaki Japan; 3 Ako Municipal Hospital Ako Japan; 4 Mixed Methods Program and Department of Family Medicine University of Michigan Ann Arbor, MI United States

**Keywords:** communication, mixed methods case study research, online social networking, smoking cessation, smoking relapse

## Abstract

**Background:**

Smoking relapse prevention after completion of a smoking cessation program is highly germane to reducing smoking rates.

**Objective:**

The purpose of this study was to evaluate the 1-year outcomes of a social media–based and peer and clinician-supported smoking cessation program on Facebook and examine communication patterns that could support smoking cessation and identify risk of relapse.

**Methods:**

We used a mixed methods case study evaluation approach featuring a single-case holistic design. We recruited volunteers who signed up after successful completion of a 12-week clinical smoking cessation program in a general medicine department in Japan. Participants contemporaneously accessed a closed Facebook page, and we analyzed their posts including text and emoticons. We used joint display analysis, which involved iterative structuring and restructuring construct-specific tables with both types of data to find the most effective approach for integrating the quantitative results with the qualitative results of content analysis.

**Results:**

One successful participant and 2 relapsed participants were analyzed to explore the specific patterns of postings prior to relapse. Decisive comments about quitting smoking were common among participants, but encouraging messages for peers were more common from the successful participant. Comments seeking social support and reassurance were warning signs of relapse. Conflicted comments also may be a warning sign of relapse risk.

**Conclusions:**

These findings based on a mixed methods case study of a social media platform supporting smoking cessation could be used to guide messaging in other online social networking service communities after a smoking cessation program to help reduce smoking relapse.

**Trial Registration:**

UMIN Clinical Trials Registry UMIN000031172; https://upload.umin.ac.jp/cgi-open-bin/ctr/ctr_view.cgi?recptno=R000035595

## Introduction

Smoking rates in developed countries are generally decreasing as a consequence of the multidimensional approaches of governments and private sector initiatives. In 2017, the overall smoking rate in Japan was 17.7%, which was higher than the 2016 rate of 15.8% in the United Kingdom and 15.5% in the United States [1-3]. Cigarette smoking is estimated to cause about 1 of every 5 deaths in Japan and the United States each year compared to about 1 of every 6 deaths in the United Kingdom [2,4,5]. The Ministry of Health, Labour, and Welfare (MHLW) in Japan set an aim of achieving an overall smoking rate of less than 12% by the end of 2022 [6]. Several communication methods, such as mobile phone- and email-based counseling, have been found effective for supporting cessation [7]. However, estimates from the MHLW suggested that about 70% of people who quit smoking relapsed in the first year following smoking cessation in Japan [8]. Therefore, reducing the rate of relapse after completion of a smoking cessation program remains an important need for achieving Japan’s national aim of smoking reduction.

Various interventions for relapse prevention have been attempted, and only extended pharmacotherapy with varenicline has been effective based on a moderate certainty of evidence [9]. Results from the National Epidemiologic Survey found that significant predictors of relapse included the first year timeframe after attempting to quit and younger age at the time of smoking cessation; the probability of relapse decreased over time [10]. These findings suggest there is a special need to prevent smoking relapse within the first year of quitting. Few studies have been designed to develop effective interventions to prevent smoking relapse after smoking cessation programs, and novel strategies are needed. At the home institution of this study, a smoking cessation outpatient clinic was established in 2011. Clinical operations originally were organized by a cardiologist and a nurse, and in 2015, operations were transferred to general medicine clinicians specialized in behavioral change interventions. The primary concern of the team members was the challenge of smoking relapse 1 year after completion of the clinical program. Among participants in the program, 70% had achieved a satisfactory rate of smoking cessation upon completion of the 12-week program. Previous studies have suggested that popular social media platforms such as Facebook and Twitter might be effective in supporting smoking cessation [11]. According to the US Department of Health and Human Services, individual, group, and telephone counseling are effective treatment, and their effectiveness increases with treatment intensity [12]. The guidelines advocate two components of counseling that are effective and recommend that clinicians should use these when counseling patients making a quit attempt: practical counseling (problem solving/skills training) and social support delivered as part of treatment. Online social networking services have the potential to address both of these key components [12]. Thus, our clinical research group sought to prevent smoking relapse after contemporaneous completion of a smoking cessation clinic program through peer counseling with clinician support on a social media platform.

**Figure 1:**
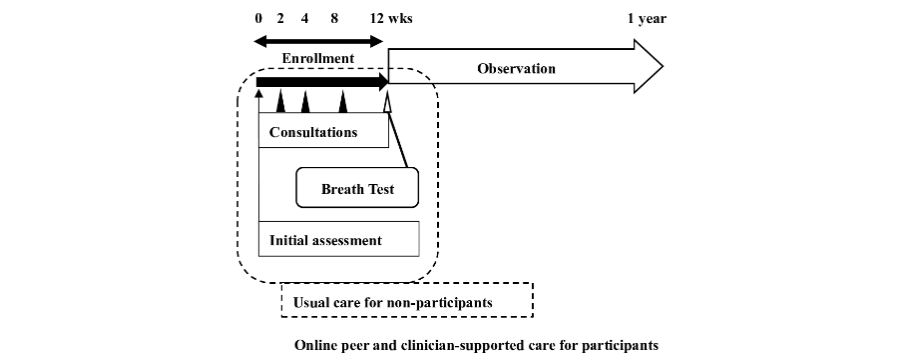
Overview of study procedures. The 12-week clinical smoking cessation program included up to 5 consultations. Original smoking cessation clinic program ended at the final consultation as indicated by the dotted square.

Due to the recent increase in online activity, we posited that online social networking services might contribute to the maintenance of smoking cessation after the completion of a smoking cessation program [13]. As an extension of a 12-week clinic-based smoking cessation program, we created a platform on Facebook as an intervention for peer and clinician-supported smoking cessation (Figure 1). The platform was the result of a clinical pilot study that showed encouraging findings. The aims of this study were to evaluate the 1-year outcomes of this peer and clinician-supported smoking cessation program that used Facebook and identify patterns that supported smoking cessation or, conversely, were associated with relapse during the 1-year observational period after smoking cessation.

## Methods

### Design

We conducted a mixed methods case study evaluation approach featuring a single-case holistic design [14-16]. We made this choice as we were examining a single program, and while the data were appropriate for an in-depth analysis, data were only available on a small number of participants. We bounded the case evaluation to the 1-year period of April 2018 to March 2019 within an ambulatory general medicine department in Japan. Relevant stakeholders included clinical physicians, staff, and all participants who received counseling during a 12-week clinical smoking cessation program including 3 participants who joined the online Facebook-based smoking cessation app. This study was approved by the regional ethics review board at Ako City Hospital (AkoHospital2017-0021).

### Setting

A smoking cessation outpatient clinic at an urban hospital operated by general physicians, nurses, and pharmacists served as the setting for this research. The clinic is located in a city with a population of 47,000 in Hyogo Prefecture in Western Japan. Serving roughly 20 patients per year, the appointment-only smoking cessation clinic was staffed by 3 physicians who had more than 3 years of experience each and 16 years among them in smoking cessation treatment. In the program, patients could choose either oral varenicline or nicotine patches in light of indications for their medical treatment. The treatment program was structured such that physicians provided individual patient consultations at the clinic 5 times over 12 weeks as a time frame that ensured coverage by medical insurance (Figure 1).

### Sample

The target population for this study was adult patients who had made an appointment for smoking cessation in the clinic. The primary inclusion criteria were an interest in participating in the program and having a smartphone. Patients younger than 20 years or older than 69 years were excluded from the study. Eligible patients were told they could begin participating in the Facebook support program during any of their visits to the smoking cessation clinic, and the study was explained to them. This arrangement allowed individuals who were unfamiliar with Facebook time to practice using the app before a subsequent visit. Among individuals indicating interest, written consent for participation was obtained by the researchers. Participation in the Facebook group was limited to participants who started abstinence. Abstinence was confirmed by interview and breath test for each visit during the clinic program. After registering on the social media site, participants could access the dedicated Facebook platform. Smoking cessation program participants could opt in or out of participating in the Facebook platform without restriction.

### Intervention

Our smoking cessation group on Facebook was developed by one of the team researchers. The program was designed to allow users who had Facebook on their smartphones to post comments, photos, and videos. The use and browsing of the Facebook links were limited to registered participants. Contributors included (1) registered patients, (2) physicians in charge of the smoking cessation outpatient clinic, and (3) clinic nurses. This approach ensured the development of a closed Facebook group. Individual identities were concealed from everyone on Facebook, including all contributors’ friends. The privacy of participants was secured using a setup feature on the Facebook app to create a closed community for our program. Staff members were expected to help patients who struggled with the temptation to smoke by using several communication methods such as reflective listening to show attention to the participants by responding to posted messages and confirming understanding of participants’ ideas and by showing empathy. Successful abstainers were encouraged to support others by offering advice based on their own experiences of coping with difficult situations and maintaining smoking cessation. The Facebook intervention was designed to provide the convenience of peer and clinician access anywhere and anytime to support patients during and after the smoking cessation clinic program. The content of each thread was monitored by each physician using the flag system function on the Facebook app. Our clinic team members strived to respond to any posts or questions based on the transtheoretical model on the same day they were posted by the participants. If there were no postings for several days, the clinical team posted relevant information such as tobacco toxicity, relaxation techniques, and greeting messages.

### Theory Underlying the Intervention

The transtheoretical model provides a widely used guide for the development of interventions for high-risk populations to change multiple health risk behaviors [17]. The constructs of the transtheoretical model are composed of stages and processes of change, decisional balance, and self-efficacy or temptation. The transtheoretical model was used by study clinicians to support smokers in the clinic to help them progress through the stages of change to tailor interventions to individual needs at each stage of change. The stages of change include 6 constructs: precontemplation, contemplation, preparation, action, maintenance, and termination. Moreover, there are 10 constructs in the process of change in this model: (1) consciousness raising, (2) dramatic relief, (3) self-reevaluation, (4) environmental reevaluation, (5) self-liberation, (6) helping relationships, (7) social liberation, (8) counterconditioning, (9) stimulus control, and (10) reinforcement management. To analyze the participants’ posts, we focused on the specific constructs relating to each stage: self-reevaluation in the preparation stage; self-liberation in the action stage; and helping relationships, counterconditioning, stimulus control, and reinforcement management in the maintenance stage.

We assumed that participants in the smoking cessation outpatient clinic were in the action stage because they had made an appointment to quit smoking. In the action stage, participants’ smoking cessation experiences were supported with medical treatment until the fifth and final consultation when smoking abstinence was confirmed in a clinical interview with an exhaled carbon monoxide test (Picoplus Smokerlyzer, Harada Corp). As our participants proceeded from the action stage to the maintenance stage, our clinical care and app-based intervention was guided by the transtheoretical model theory and entailed providing social support and reassurance to help participants overcome any relapse crises. We designed the research to observe patterns in the posts on the app that supported smoking cessation, or, conversely, pointed to a smoking relapse during the action and maintenance stages. The clinician researchers staffing the clinic and hosting the Facebook platform developed a shared understanding of the transtheoretical model through a general medicine department lecture-discussion training. They also used a counseling approach based on the transtheoretical model. They routinely incorporated these strategies to motivate patients who needed clinical smoking prevention support. By extension, the clinical research team were readily able to discern Facebook posts reflecting constructs in the model.

### Data Collection Procedures

#### Quantitative Data Collection

Data on participant demographics, confidence to quit smoking, and the Fagerstrom Test for Nicotine Dependence (FTND) were obtained at the initial clinic visit [18]. The duration of smoking cessation was confirmed by self-report in a follow-up telephone interview by a smoking cessation clinic physician. Relapse was defined when the participant self-reported any puff of smoking behavior and relapsed with continued smoking. Each participant’s number of posts was counted at the end of the study.

#### Quantitative Data Analysis

Demographic data, confidence to quit smoking, FTND, and duration of smoking cessation were compared between participants and nonparticipants to examine for patterns. The number of postings, codes, and categories were counted and compared between participants.

#### Qualitative Data Collection

Typed comments and 3 types of emoticons: typographic face marks (small-size illustrations added at the end of words or sentences, eg, #^.^#), nonlinguistic symbols (eg,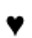), and colorful inline graphics (eg, emoji 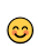) submitted by participants on the Facebook discussion board were downloaded and saved in Word (Microsoft Corp) by the authors for analysis [19]. The research team organized the data in chronological order for each participant.

#### Qualitative Data Entry and Analysis

We used a combination of deductive coding based on the elements of the transtheoretical model and inductive coding as our team allowed for emerging codes from the full range of data sources from the participant postings [20]. Similar methods have been used to analyze social media content from Facebook, Twitter, and YouTube [21]. The content of the comments and responses from our Facebook platform were coded by two independent researchers using MAXQDA 2018 (VERBI GmbH). Discrepancies in their coding were resolved through group discussion among the team researchers who reviewed the original data to interpret the context. Similar codes were merged into categories reflecting the underlining meaning of the original data. Analytical rigor was achieved through attention to credibility, dependability, and confirmability [16]. For credibility, the research team spent time immersed in the original data and discussing the underlying meanings of posted comments. Dependability was achieved through the use of data code-recode procedures. Confirmability was achieved by recording notes in the extracted codes. We also performed content analysis of the emoticons attached to the textual comments and responses.

The participants’ typed data and emoticons were analyzed descriptively to examine their influences on smoking cessation. After coding the qualitative comments of each participant, frequency analysis was conducted to examine the distribution of codes for each participant using MAXQDA 2018. The types of emoticons were analyzed as independent codes simultaneously. Additionally, MAXMaps, one of the graphic functions of MAXQDA, was used to explore connections among the different elements of the codes visually in a workspace as a map; the goal was to find deeper relationships among the codes. The primary outcome was a description of factors contributing to smoking cessation among the participants based on a comparison of patterns in the MAXMaps between the successful and relapsed participants.

### Mixed Methods Joint Display Analysis

We used an interactive approach in the joint display analysis to arrive at a deeper understanding of the results [22]. The analysis involved structuring and restructuring a table by juxtaposing both types of data to find the most effective approach for integrating the quantitative results with the qualitative results of the content analysis [23,24]. First, the frequencies of categories in the text were summarized for each case and linked with the relevant quotes that had produced these categories. Second, we reorganized the master table based on the frequency of posts, from the most often to the least. Third, we created a new joint display to compare the successful abstainer (participant 1) and the 2 who relapsed (participants 2 and 3). Fourth, we redesigned the table to include a classification of category types according to the transtheoretical model, an interpretation of the quantitative frequency counts, and the qualitative characteristics of the comments. Through this process, we identified 3 primary categories of comments: helping relationships (7 types), self-liberation (1 type), and self-reevaluation (2 types). Last, we divided the very long master table into 2 tables featuring (1) helping relationships and (2) self-liberation and self-reevaluation.

## Results

### Sample

A total of 13 people attended the clinic during the study period. Four patients were ineligible due to hospitalization (n=2) and dropout (n=2) from the study. The remaining 9 patients were deemed eligible as they committed to quitting smoking at the initial visit, and all 9 were invited to use the social media peer support group. Three joined while 6 declined. The 3 Facebook group participants entered the peer and clinician-supported smoking cessation program after confirmation of quitting smoking by a clinical interview and exhaled carbon monoxide test. As they attended the clinic program individually, they entered the study at slightly different times and did not know each other. However, their participation in the study writing posts and responding to posts from the peers and staffs were contemporaneous (Multimedia Appendix 1).

### Quantitative Findings

The characteristics of the Facebook abstention support platform participants were compared to the nonparticipants and noneligible patients (Table 1). The total number of postings by physicians, nurses, and 3 participants was 33, 13, and 43, respectively. The study analysis focused on the interactions between the participants and between the clinicians and participants. We also observed the participants communicating with each other by posting and leaving emoticons.

**Table 1:** Characteristics of Facebook participants compared to non-Facebook participants and noneligible patients during the study period.

Variable			Eligible				Noneligible (n=4)
			Participant (n=3)		Nonparticipant (n=6)		
**Gender, n (%)**							
	Male	3		4		3	
	Female	0		2		1	
Age (years), mean (SD)			55.6 (6.0)		51.7 (13.5)		49.8 (16.6)
Confidence^a^ (%), mean (SD)			77 (25)		62 (28)		50 (0)
FTND^b,c^ (points), mean (SD)			5.0 (1.7)		6.2 (2.4)		6.3 (1.5)
Duration of smoking cessation (month), mean (SD)			5 (6.1)		4.5 (3.5)		—^d^

^a^Percentage of confidence to succeed in smoking cessation and FTND were obtained at the first encounter at the smoking cessation outpatient clinic.

^b^FTND: Fagerstrom Test for Nicotine Dependence.

^c^FTND level of nicotine dependence interpretation: low (0-3), middle (4-6), and high (7-10).

^d^Not applicable.

### Successful Smoking Cessation and Relapse

Based on a telephone confirmation conducted at 1-year follow-up after completing the smoking cessation program, one patient (participant 1) had succeeded in smoking cessation. Two patients had relapsed: one (participant 2) at 2 months, and the other (participant 3) at 1 month after completing the smoking cessation clinic program (Table 2). Confidence in successful smoking cessation at baseline was 80% in participant 1, 50% in participant 2, and 100% in participant 3. The FTND at the initial visit showed a low-level nicotine dependence in participants 1 and 3, and a moderate level in participant 2. We illustrate schematically the time course for each participant in Multimedia Appendix 1.

**Table 2:** Demographics of the 3 participants who joined the peer support Facebook group.

Variable	Participant 1	Participant 2	Participant 3
Outcome	Success	Relapse	Relapse
Age	62	50	55
Gender	Male	Male	Male
Confidence (%)	80	50	100
FTND^a^ (points)	4	7	4
Months continuing smoking cessation after the clinic program	12	2	1

^a^FTND: Fagerstrom Test for Nicotine Dependence.

### Qualitative Findings

We used 11 codes for the coding scheme. The codes and their relationships to the transtheoretical model are presented in Table 3. The distribution of codes fell primarily into 3 constructs in the process of change. The codes assigned to the category helping relationships were gratitude, special gratitude (indicating intensity of gratitude through repetition of words or emoticons), one’s updates, encouragement, agreement, anticipation of support, one’s own ideas for smoking cessation, and health problem consultations. The codes assigned to the category self-reevaluation were worry and happiness during smoking cessation. The code assigned to self-liberation was the participant disclosing the decision to quit to others.

According to the processes of change in the transtheoretical model, helping relationships is described as caring, trust, openness, and acceptance as well as support from others for healthy behavior change (eg, a positive social network) [17]. The code own ideas of maintaining smoking cessation belongs to the helping relationships in Table 3. The quotes described in Table 4 show participant views such as “Smoking cessation 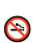 has a negative image. I think we need to change the image of smoking cessation into positive one (#^.^#).” This participant proposed his idea to maintain abstinence for other participants, leading to making new relationships among peers to help each other. There were no postings relating to other constructs in the maintenance stage of the transtheoretical model such as counterconditioning, stimulus control, and reinforcement management.

**Table 3:** Coding scheme and its relationship to the transtheoretical model.

Code name	Explanation	Corresponding construct in the transtheoretical model
Gratitude	Expressing gratitude toward staff of the smoking cessation clinic. It can strengthen the relationship between participants and staff to focus on successful smoking cessation.	Helping relationships
Special gratitude	Expressing special gratitude toward staff.	Helping relationships
One’s updates	A report of one’s present smoking status. Participant acknowledges their smoking cessation or relapse. Being honest about oneself can strengthen the mutual relationships between participants and staff.	Helping relationships
Encouragement	Posting encouraging messages to peers aimed at sustaining smoking cessation.	Helping relationships
Agreement	Agreeing with peer’s ideas relating to smoking cessation.	Helping relationships
Anticipation of support	Anticipating the staff tracking one’s smoking cessation, and giving advice if necessary for support.	Helping relationships
Own ideas for smoking cessation	Own ideas of maintaining smoking cessation such as rebuilding a positive image of smoking cessation to aim for success.	Helping relationships
Health problem consultation	Asking for advice from staff about one’s health condition during smoking cessation.	Helping relationships
Worry	Commenting on concern about continuing smoking cessation.	Self-reevaluation
Happiness during smoking cessation	Expressing one’s happiness about discovering positive results of smoking cessation.	Self-reevaluation
Decision	Announcing one’s decision to quit smoking formally just before starting smoking cessation based on one’s belief.	Self-liberation

**Table 4:** Smoking cessation and helping relationships from the transtheoretical model^a,b,c^.

Categories (# comments)	Smoking cessation success		Smoking relapse							Overall interpretation (meta-inferences)
	P1^d^		P2^e^		P3^f^		P2, P3			
	n (%)	Illustrative quotes	n (%)			Illustrative quotes				
Expressions of gratitude (n=9)	6 (22)	*The staff have been wonderful. (#^.^#)* *After all, your “backup” had a profound effect on me. (#^.^#)* *Thank you.* 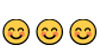	3 (17)	0		*Thank you. [P2]* *Thanks for your advice. [P2]*		There were more frequent and more descriptive comments from successful (P1).		
Reports about smoking cessation status (n=9)	5 (19)	*So far, so good, but I’m not so confident, so please give me your support and advice.* （ 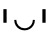 ） *Well, I’m doing rather well.* *I’m trying to spend more days without the pill. So far, I’m keeping smoking cessation.* （ 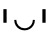 ） *Two weeks have passed since April 9th when I stopped taking Champix, just before the 10th week of treatment. I’m doing well keeping smoking cessation.* （ 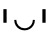 ） *Thank you, I’m still quitting smoking.*	2 (11)	2 (50)		*One week has passed since quitting smoking. [P2]* *Somehow I got through it. [P2]* *This is the second clinic. I have been quitting smoking for 1 week. [P3]* *I got it how to post. Now, I am somehow still quitting smoking. [P3]*		Both the frequency and meanings of the comments were similar among all participants.		
Encouraging messages (n=9)	7 (26)	*Let’s do our best!* （ 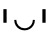 ） *Try your best.* 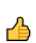 (#^.^#) *If you quit smoking, you will find you can rediscover various smells around you.* （ 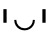 ） *I’m on your side.* (#^.^#) *As there are fewer smoking-allowed areas, the price has risen, now is a chance to quit smoking!*	2 (11)	0		*Let’s do our best together. [P2]* *Yes, let’s try our best. [P2]*		There were more frequent and more descriptive comments from successful (P1).		
Agreement with other’s comments (n=4)	2 (7)	*I agree (#^.^#). We are taking a good challenge, but [smoking cessation] is stigmatized negatively.* (#^.^#)	2 (11)	0		*Hmmm, motivation. I will try to find it. [P2]* *Carbon monoxide concentration, which we had examined before when seeing the doctor. [P2]*		Frequency and descriptive comments were similar between successful (P1) and relapse (P2).		
Anticipating support of smoking cessation from staff	1 (4)	*Please watch over me from Tottori.* (#^.^#)	0	0		—^g^		This category was specific in successful (P1).		
Own ideas for continued smoking cessation (rebuilding positive image of smoking cessation)	1 (4)	*Smoking cessation* 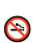 *has a negative image. I think we need to change the image of smoking cessation into positive one.* (#^.^#)	0	0		—		This category was specific in successful (P1).		
Asking advice about own health	0	—	1 (6)	0		*Good evening. I don’t know if it is a problem related to tobacco, but I’d like to ask you about difficulty sleeping. [P2]*		This category was specific in relapse (P2).		

^a^Comparison of the participants with smoking cessation success and relapse using the frequency of comments and illustrative comments using a joint display.

^b^Helping relationships refer to the process of change that mediates progression in the maintenance stage.

^c^The numbers of each cell represent frequencies of codes with percentage of all codes noted in brackets in each case.

^d^P1: participant 1.

^e^P2: participant 2.

^f^P3: participant 3.

^g^Not applicable.

### Focus on Helping Relationships on the Platform

The vast majority of comments posted on the platform from the participants concerned helping relationships. A comparison of the decreasing order of frequencies and illustrative comments for the successful abstainer and the 2 participants who relapsed are provided in Table 4. A total of 7 categories relating to helping relationships were identified through the coding scheme. The initial coding scheme differentiated between gratitude and special gratitude as participants clearly used these to express the degree of emphasis that they symbolized by lining up several kinds of emoticons, such as (#^.^#) and 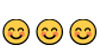. These 2 codes were aggregated into a single category named expressions of gratitude. In terms of the frequency and percentage of comments posted on the platform, encouraging messages were specifically observed in the successful participant 1, while reports of smoking cessation status were observed similarly among all 3 participants. Encouraging messages posted by the successful participant 1 were more descriptive than those of the relapsed participant 2.

The percentage of categories referable to helping relationships was 81% (22/27) in the successful participant 1, 56% (10/18) in relapsed participant 2, and 50% (2/4) in relapsed participant 3 (Table 4).

### Self-Liberation and Self-Reevaluation on the Platform

Of the categories in the transtheoretical model, self-liberation was more commonly mentioned. There were 2 categories of self-reevaluation (Table 5). The category decision to stop smoking was observed in all participants with similar descriptive comments. Notably, participant 2 mentioned most frequently concern about sustaining smoking cessation in his comments. In addition, the category expressing happiness about the successful result of smoking cessation was specific to participant 2. Content analyses of the emoticons attached to textual comments, and responses showed that participant 1 used them 30 times during the study period. They were especially common in his expressions of gratitude and encouraging messages. Participants 2 and 3 used only a few emoticons, thus precluding deeper analysis.

**Table 5:** Smoking cessation as relates to self-liberation and self-reevaluation based on the transtheoretical model^a,b,c^.

Constructs of processes of change and categories (total number of comments made)		Smoking cessation Success		Smoking relapse			Overall interpretation (meta-inferences)
		P1^d^		P2^e^	P3^f^	P2, P3	
		n (%)	Illustrative quotes	n (%)		Illustrative quotes	
**Self-liberation**							
	Decision about smoking cessation (n=7)	2 (7)	*I’ll do my best, and say goodbye to tobacco forever.*（ 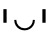 ） *I’ll do my best not to fail expectations.*（ 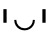 ）	3 (17)	2 (50)	*Looking ahead, I’ll try to continue smoking cessation, so please give me your support.* [P2] *I am going to do it.* ＼(^o^)／ [P2] *I’ll try.* (P2) *I will do my best.* (P3) *The 3-month study*^g^ *is over. I received a certification of having quit smoking. This is the start of the struggle within myself. If I fail, I will be back for support again in a year.* ^O^ [P3]	The frequency was higher in relapse (P2 and P3), but descriptive comments were similar among (P1), (P2), and (P3).
**Self-reevaluation**							
	Concern about sustaining smoking cessation (n=4)	1 (4)	*I worry about (doing too well after quitting smoking).*	3 (17)	0	*Today I feel 70% like smoking, what should I do?* [P2] *I have been in the building all day long, so I feel like going out.* [P2] *It was very difficult to improve the [carbon monoxide] value from 1 to 0.* 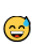 *Every time, I had to laugh wryly with the nurse.* 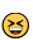 *Next time, I will achieve 0. Maybe* 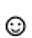 . [P2]	The frequency was higher and more descriptive comments were observed in relapse (P2).
	Expressing happiness about positive result of smoking cessation (n=1)	0	—^h^	1 (6)	0	*Ah! Oh yeah, I changed my toothbrush and toothpaste. I feel better because I was annoyed by tar and coffee stains.* 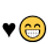 [P2]	This category was specific in relapse (P2).

^a^Comparison of the participants with smoking cessation success and relapse using the frequency of comments and illustrative comments in a joint display.

^b^Self-liberation is the process of change that is seen in the action stage while the category self-reevaluation refers to the process of change that mediates progression between the contemplation and preparation stages.

^c^The numbers of each cell represent the frequencies of codes with the percentage of all codes noted in brackets in each case.

^d^P1: participant 1.

^e^P2: participant 2.

^f^P3: participant 3.

^g^The participants used the phrase “referred to the smoking cessation clinic program.”

^h^Not applicable.

### Longitudinal Findings for Successful Smoking Cessation and Relapse

**Figure 2:**
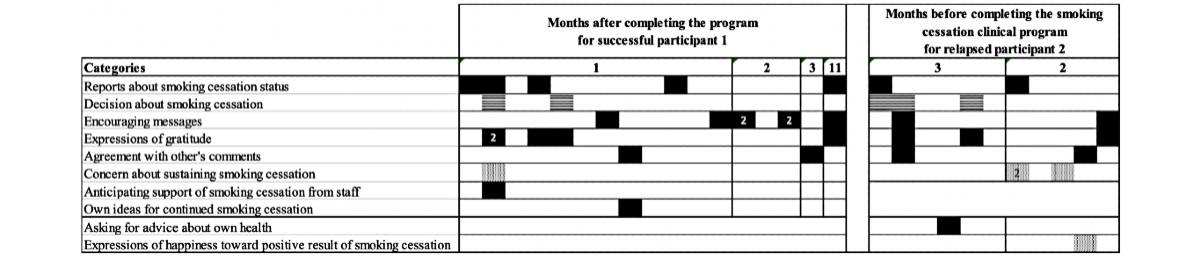
Categories of comments appearing by successful participant 1 and relapsed participant 2 during the observed period. Boxes represent the appearance of each category in time sequence after the participant completed the smoking cessation program at the clinic. Filled, striped, and dotted boxes represent categories corresponding to helping relationships, self-liberation, and self-reevaluation, respectively. The number in the box represents the frequency of the observed category. Boxes without numbers represent 1 observed occurrence.

The transitions between major categories in the transtheoretical model during the study period in participant 1 and participant 2 are demonstrated in Figure 2.

### Evolution of Comments Made by Participant Who Succeeded in Smoking Cessation

Participant 1 commented on the decision of smoking cessation twice during the first month after starting the peer and clinician-supported smoking cessation program. This category never appeared after that time. It was followed by 7 encouraging messages toward peers (Figure 2). Afterward, other categories corresponding to the maintenance stage accounted for the comments.

### Evolution of Comments by Participants Who Relapsed After Smoking Cessation

Participant 2 joined in the Facebook abstention support platform after the first visit of the 12-week clinical smoking cessation program. He quit smoking at the initial visit and sustained smoking cessation with medical treatment by oral varenicline. In contrast to participant 1, participant 2 wrote encouraging messages only twice after his decision to stop smoking. He asked for advice on managing insomnia, which was followed by concerns about sustaining smoking cessation 3 times (Table 5, Figure 2). Notably, he expressed happiness toward a positive result of smoking cessation, and especially, restoration of oral hygiene. These categories were regarded as one of the constructs in the processes of change, self-reevaluation, generally seen in the stages of contemplation or preparation. On the other hand, he posted encouraging messages around the same time. In short, the categories belonging to both the stage of contemplation (or preparation) and the stage of maintenance were observed around the same time (Figure 2). The categories referable to self-reevaluation accounted for only 4% (1/27) in the successful participant, while these constituted 22% (4/18) in relapsed participant 2 (Table 5). Three months after his last comment, he relapsed into smoking as confirmed by a follow-up telephone interview. In short, participant 2 retreated from the maintenance stage after completing the smoking cessation clinic program.

Participant 3 also quit smoking when he visited the clinic for the first time. Because he needed time to learn how to access the page, he joined the online smoking cessation program 2 weeks after his first visit. Participant 3, who also relapsed, posted 4 comments during the study period. Similar to participant 2, he posted only 2 comments referable to the category decision to stop smoking (Table 5). The comments were related to self-liberation just after completing the smoking cessation clinic program. The participant did not post any encouraging messages (Table 5). He relapsed to smoking 1 month after his last post.

### Repetitive Posting of Encouraging Messages

**Figure 3:**
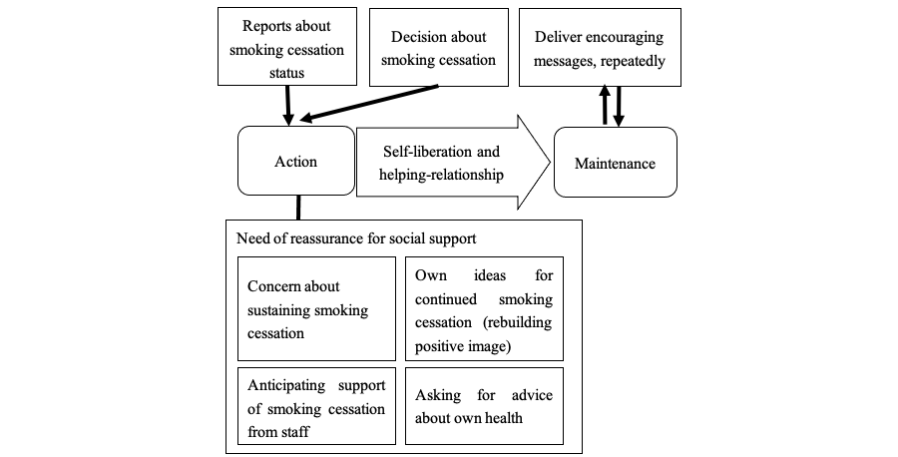
The repetitive encouraging message model for smoking cessation aligned with stages in the transtheoretical model. Square boxes: categories identified in this study. Rounded boxes: stages of change. Arrow: constructs in the process of change.

Based on the stages of change in the transtheoretical model, we integrated the categories of our results into a conceptual framework (Figure 3). In the transtheoretical model, self-liberation is necessary for the process of change from the action stage to the maintenance stage [17]. We interpreted the category of decision about smoking cessation to represent self-liberation. Concern about sustaining smoking cessation is considered as a category in self-reevaluation, usually seen in the preparation stage.

The relationships between processes and stages have not been strictly consistent [17]. Therefore, this category was interpreted as one of the needs of reassurance for social support as well as other 3 categories in helping relationships: anticipating support of smoking cessation from staff, own ideas for continued smoking cessation, and asking for advice about own health. Additionally, we observed repeated encouraging messages as helping relationships, which were necessary for maintaining smoking cessation. We propose a repetitive encouraging message model as highly important to the maintenance stage of smoking cessation.

## Discussion

### Benefits of the Smoking Cessation Program

Regarding the first study aim of evaluating the 1-year outcomes of the smoking cessation program, the findings were lackluster. Of the 9 who met eligibility criteria for study, 3 had quit smoking after participating in the smoking cessation clinical program. Of these 9, 3 participants chose to participate in the Facebook-based peer and clinician-supported platform and only one persisted in smoking cessation to the 1-year end point. Among the 6 nonparticipants in the original clinical smoking cessation program, 2 successfully quit smoking. The social media platform intervention outcome proved no better than the nonintervention group. According to national data from Japan, about 70% of people who quit smoking relapse in the first year after smoking cessation [8].

### Possible Explanations for Low Enrollment on the Platform

Regarding decision making about whether to enroll in the program, it is plausible that the least confident patients would tend to enroll in the online program. If true, it is possible that the intervention might have increased the success rate of smoking cessation compared to having no intervention. Regarding the nonparticipants, 2 of the 6 refused enrollment due to concerns about insufficient security of personal data on Facebook. This study was ongoing when the leakage of private data of Facebook users was reported widely in the media in Japan, as elsewhere [25]. This issue almost certainly negatively affected eligible participants decision making about participation in the study. Lack of trust and confidence in security of the platform could obviously limit the use of this method in the future.

### Plausible Reasons for Limited Number of Sustained Smoking Quits

There are several plausible reasons why a higher rate of sustained smoking cessation success was not achieved. First, the idea of a social media platform for support of smoking cessation, at least for the demographic in this region, may not have been sufficiently appealing. This could be related to the demographic of participants as older individuals, who are more likely to be smokers. Being older, they may also have been less facile with technology and social media platforms. Interestingly, participant 1, who successfully quit, was aged 62 years during the study and extensively used the platform for text and emoticon postings. Hence, caution should be exercised in assuming that being older precludes participation in social media. Second, the benefits of the platform may have been suboptimal as there was limited time available for usability testing. It is encouraging that all 3 participants who started on the platform remained engaged for several months. Third, achieving greater success on a platform such as the one evaluated may require a critical mass. Three people may not suffice, especially if only one provides positive comments. With a larger number of participants, interactivity between multiple subscribers may have demonstrated more beneficial effects. Fourth, the optimal involvement of program staff is unknown. It is notable that some comments were directed to the health professionals who were known by the platform users. We did not collect data on participants’ views of clinic program staff involvement.

### Patterns Supporting Smoking Cessation or Related to Relapse

Our second study aim was to identify patterns supporting smoking cessation or related to relapse. We observed that repetitive encouragement offered to peers during the early smoking cessation period appeared to help solidify the maintenance stage, as seen in participant 1 but not in participant 2. On the other hand, the need for reassurance through social support such as in the form of concern about sustaining smoking cessation was in hindsight an important warning sign of smoking relapse risk. Conflicting comments, such as encouragement for others while endorsing annoyance with concerns about sustainability of smoking cessation, might be a potential warning sign as there were both positive and negative feelings at the same time. Smoking relapse is caused by various factors such as withdrawal symptoms, negative affect, and cravings [26]. We observed negative feelings and craving during the second month of enrollment in participant 2. The participant made encouraging comments and expressed gratitude and happiness about a positive result of smoking cessation during this tough time. But, in hindsight, he was struggling to maintain abstention. Emotional distress has been reported as an immediate precursor factor in smoking relapse in a longitudinal study [27]. While encouraging messages were sent by medical staff soon after he posted comments, if his conflicted feelings had been noted, more active management might have been possible.

### Utility of Social Networking Services to Send Messages Helpful to Support Smoking Cessation

The effectiveness of behavioral change through mobile health software apps has been noted in terms of perceived psychological empowerment and enhanced hedonic well-being [28]. The development of apps to discriminate among text messages could focus on distinctions among the decision of smoking cessation, encouragement for peers, and need for social support such as concern about sustaining smoking cessation. It could alert health professionals supporting patients within the peer group to intervene quickly, or it could automatically reply with helpful messages for those participants. Mobile phone users with these apps appreciate the time-sensitive aspect of such devices. A systematic review has reported that social support is a partially efficacious method for quitting smoking [11]. It is not feasible to continue to respond manually to every post from participants when the number of platform users increases beyond the capacity of the clinical team to manage. Natural language processing or other artificial intelligence applications could be used in the future for analyzing the contents of posts from the participants who struggle with maintaining tobacco cessation. Automatic responses to messages from abstainers could help health professionals support patients trying to quit smoking on a nationwide scale. The real-time messaging could provide advantages of human staffing which is difficult 24/7.

### Study Findings in a Broader Context

The potential benefits of mobile phone-based smoking cessation interventions have been reported in a systematic review [7]. Text message–based smoking cessation interventions, either alone or in combination with face-to-face assessments or online programs, were effective for those trying to quit smoking in high-income countries with existing tobacco control policies, media, and education [7]. We used a similar strategy involving text message–based support on a Facebook group page. In another study using Twitter-based support networks for adult smoking cessation, researchers elucidated reciprocated ties among abstainers and nonabstainers in both dyadic and small-group communication patterns [29]. We found similar reciprocated comments and responses between participants 1 and 2 (data not reported, available upon request). This finding may imply that the spirit of mutual aid was shared among participants aiming for successful smoking cessation.

### Additional Lessons Learned

These findings raise the question about the utility of creating a private community on an online platform like Facebook for adult smoking-cessation interventions that support collaboration between smoking cessation-patients and health professionals. A recurring pattern among smoking cessation clinics is rapid relapse to smoking soon after treatment termination [17]. The sudden loss of social support can threaten the stability of smoking abstention. The merit of a private community is a continuous mutual relationship between staff members and patients after completing a maximum 12-week smoking cessation outpatient clinic. Such a community may ensure that participants share their feelings and ideas without shame.

Our findings about the potential utility of Facebook as a platform for smoking cessation are consistent with previous reports [30,31]. Previous research additionally has revealed potentially useful features of iPhone apps for smoking cessation that focused on exploring behavioral change techniques in apps [32]. A pilot cluster randomized controlled trial showed the possible usefulness of the social group for recent quitters who had completed an 8-week treatment and reported abstinence for at least 7 days in Hong Kong [33]. The study found significant relapse prevention at 2-month and 6-month follow-ups in users allocated in the WhatsApp group but insignificant results in the Facebook group. They also analyzed the posts and collected participant postintervention feedback [34]. They suggested that they could improve the online peer support group’s effectiveness by encouraging more self-report of relapse, active discussions, sharing of interesting content, and using an appropriate discussion platform.

The main components of behavioral change techniques for smoking cessation include supporting identity changes; rewarding smoking cessation; and offering advice on changing routines, coping strategies, and medication use [35]. If an app such as an automatic reminder is developed using smartphones, the method and outcome should be assessed in the context of evidence-based practice. Few apps available by popular app stores are rooted in evidence-based science [36]. Future iterations of online platforms should fully consider this body of evidence.

Tobacco control challenges are universally problematic in East Asia [37]. For example, Japan, China, and the Republic of Korea face similar problems such as high rates of adult men who are current smokers, a low rate of men planning to quit smoking, and fewer opportunities to use nicotine replacement therapy than in Western countries. Younger generations in these populations are highly technophilic. Sharing the knowledge and skills of smoking cessation strategies and supporting apps may enhance smoking cessation programs and reduce tobacco-related diseases in these countries.

### Methodological Insights

In this paper, we have used a mixed methods case study design. For the intensive evaluation of the cases, the quantitative counts and qualitative reports are relevant as individual data on the participants. The actual quantitative data from the frequency of codes and constructs provided meaningful information for comparing characteristics between the successful case and relapsed cases. Additionally, this paper provides several methodological insights and novel illustrations. First, the study illustrates a qualitatively oriented mixed methods case study using a single-case holistic design [38]. However, presentation of the findings followed a quantitatively oriented approach. That is, the quantitative data provided a useful organizational structure, but the qualitative data were prominent in the analysis [39]. Second, the study illustrates the use of a qualitatively oriented longitudinal mixed methods case study. Both the quantitative and qualitative findings were used interactively and were essential for the study [40], but the qualitative data provided the greatest insight about how the participants responded to the intervention. Third, the study illustrates the integration of emoticons into qualitative data analysis. Emoticons are commonly used on social media in text messaging and platforms such as Facebook, and researchers need examples for use in their work. A previous study quantitively analyzed emoticons posted on Facebook using correlation analysis [41]. Their findings supported the feasibility and validity of studying individual emotional well-being by means of examination of Facebook profile including emoticons. Fourth, the study provides a detailed explanation of steps of the mixed methods joint display analysis that were used to integrate the findings [22]. Fifth, the study illustrates the use of the transtheoretical model in a mixed methods analysis of data [42]. Sixth, the mixed methods integration dimensions are illustrated in multiple ways [43].

### Limitations and Future Research

A key limitation of this mixed methods case study was that it was conducted in a single smoking cessation clinic in Japan. Data collection in other settings is necessary to assess the relevance and transferability of our findings. The success rate for quitting immediately after completing the smoking cessation program in our hospital has hovered at about 60%, which is slightly above the average of Japanese medical agencies [8]. Smoking relapse 1 year after completing the program is about 70% in our hospital, which is similar to trends in another report [8]. These findings suggest that we had a typical setting for this case study. As the setting for this research was a single program and the number of participants was small, a case study research design was chosen over a variable-based study approach. This enabled us to look for the critical characteristics of the cases from the midst of complicated data. Our intent was to understand the case (ie, what it is, how it works, and how it interacts with its real-world contextual environment) [15]. While we offer no claims about the effectiveness of the program or generalizability, we feel the in-depth analysis of this case study using systematic procedures provided important insights that might not have been feasible to explore in experimental studies and that the findings have relevance, namely, transferability, to other settings [15]. Additionally, while duration of abstinence before smoking cessation intervention initiation is associated with maintenance of smoking abstinence, we did not have record of the exact date of their last cigarette. As we had comments prior to relapse to smoking but few afterwards, different research strategies may be needed for data collection designed to understand the immediate postrelapse period. Another challenge common to online research relates to the variation in participation of postings among participants. Future researchers may need to take this into account. Additionally, future research on a broader scale could consider a quasiexperimental design or an experimental design with a control group.

### Conclusions

The private Facebook page to prevent smoking relapse enabled us to communicate without meeting together in the same physical space, a factor not to be ignored given the need for alternative approaches during the COVID-19 pandemic. Decisive comments about quitting smoking were common among participants, but encouraging messages for peers primarily came from one successful person. The need for social support and reassurance, such as in the form of concern about sustainability of smoking cessation, was a warning sign of smoking relapse. In addition, conflicting comments, such as encouragement for others while admitting annoyance at worrying about sustaining smoking cessation, might be a potential warning sign. It should be regarded as occurring before the maintenance stage. Further analyses of these messages are needed to identify a significant pattern in comments and responses to prevent smoking relapse. Finally, this paper provided methodological illustrations relative to the use of a longitudinal mixed methods case study, the use of information from Facebook including text and emoticons, and a detailed explanation of the mixed methods joint display analysis.

## Appendix

Multimedia Appendix 1 Schematic illustrating the contemporaneous participation in smoking cessation clinic and Facebook program for the case study participants.

## Abbreviations

FNTD: Fagerstrom Test for Nicotine Dependence
MHLW: Ministry of Health, Labour, and Welfare in Japan
