# Supplementary material for: Evaluating Outcomes of a Social Media–Based Peer and Clinician-Supported Smoking Cessation Program in Preventing Smoking Relapse: Mixed Methods Case Study
Source: JMIR Form Res. 2021 Sep 20;5(9):e25883. doi: 10.2196/25883 (PMC8491124; doi:10.2196/25883)
Supplement: Multimedia Appendix 1 [file formative_v5i9e25883_app1.pdf]

Multimedia Appendix 1. Schematic illustrating the contemporaneous participation in smoking cessation clinic and the Facebook program for the case study participants.

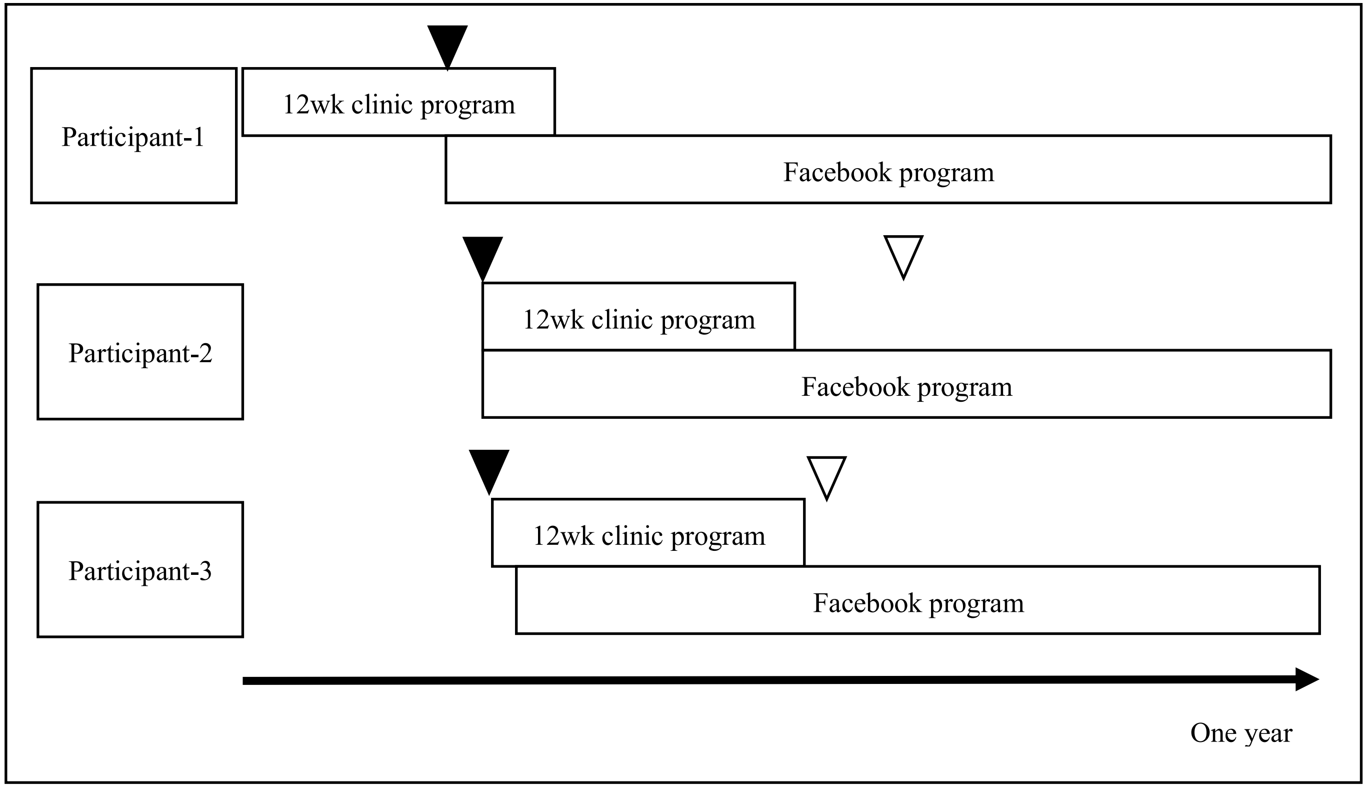

Note: The black triangles denote start of quitting smoking and white triangles indicated relapse of smoking, respectively.

Participant-1 tried quitting and relapsed smoking several times during the clinic program. He quit smoking after eight weeks of the clinic program, and then joined the Facebook program. He never relapsed during the study period. Participant-2 quit smoking when he visited the clinic the first time. He joined the Facebook program at that time. He relapsed to smoking two months after the clinic program. Participant-3 quit smoking when he visited the clinic for the first time. He joined the Facebook program two weeks after the first visit because he needed time to learn how to access to the Facebook page. He relapsed smoking one month after the clinic program. Each participant attended the clinic program individually, but they spent time contemporaneously in the Facebook program for almost same time frame.
